# Study on the correlation between triglyceride glucose index, triglyceride glucose index to high-density lipoprotein cholesterol ratio, and the risk of diabetes in nonalcoholic fatty liver disease

**DOI:** 10.3389/fendo.2025.1594548

**Published:** 2025-06-23

**Authors:** Tao Sun, Jun Liu

**Affiliations:** ^1^ Department of Hematology and Oncology Laboratory, The Central Hospital of Shaoyang, Shaoyang, Hunan, China; ^2^ Department of Scientific Research, The First Affiliated Hospital of Shaoyang University, Shaoyang, Hunan, China

**Keywords:** non-alcoholic fatty liver disease (NAFLD), triglyceride glucose index (TyG), TyG to high-density lipoprotein cholesterol ratio (TyG/HDL-c), diabetes mellitus, restricted cubic spline (RCS), subgroup analysis, risk prediction

## Abstract

**Background:**

This study seeks to investigate the association between the triglyceride-glucose index (TyG), triglyceride glucose index to high-density lipoprotein cholesterol ratio (TyG/HDL-c), and the risk of diabetes in individuals with nonalcoholic fatty liver disease (NAFLD).

**Methods:**

This retrospective study encompassed 457 NAFLD patients from The Central Hospital of Shaoyang, monitored over a three-year period. Missing data were addressed using multiple imputation, and the Synthetic Minority Over-sampling Technique (SMOTE) was employed to balance the dataset. Multicollinearity analysis was conducted to evaluate the collinearity among variables, while principal component analysis was utilized to examine the distribution of variables in both the original and balanced datasets. A multivariate logistic regression model was used to assess the association between TyG, TyG/HDL-c, and the risk of diabetes in NAFLD patients, adjusting for various covariates. Subgroup analysis was performed to identify differences across diverse populations, and restricted cubic splines (RCS) were used to explore potential non-linear relationships. The receiver operating characteristic (ROC) curve examined the diagnostic value of individual and combined indicators in assessing the risk of diabetes in NAFLD patients.

**Results:**

Upon adjustment for all covariates, TyG was found to significantly elevate the risk of diabetes among patients with NAFLD (OR = 1.96, 95% CI: 1.67-2.30, P < 0.001), with a notable non-linear relationship observed (threshold: 2.39, P-nonlinear = 0.002). Similarly, TyG/HDL-c significantly increased diabetes risk (OR = 1.90, 95% CI: 1.60-2.26, P < 0.001), also demonstrating a distinct non-linear association (threshold: 2.20, P-nonlinear < 0.001). Subgroup analyses revealed significant interactions between TyG and TyG/HDL-c across different gender subgroups (P for interaction < 0.05). The ROC curve analysis indicated that the combined use of TyG and TyG/HDL-c provided superior diagnostic performance for assessing diabetes risk in NAFLD patients (Area Under the Curve [AUC]: 0.703, 95% CI: 0.665-0.740), compared to the use of TyG (AUC: 0.694, 95% CI: 0.656-0.732) or TyG/HDL-c (AUC: 0.693, 95% CI: 0.655-0.731) independently.

**Conclusion:**

Both TyG and TyG/HDL-c are significantly associated with an increased risk of diabetes in NAFLD patients, exhibiting non-linear relationships. Furthermore, these associations vary significantly across gender subgroups, their combined use enhances risk assessment, supporting their clinical utility in evaluating diabetes risk.

## 1 Introduction

Non-alcoholic fatty liver disease (NAFLD) is a metabolic-associated hepatic disorder characterized by the abnormal accumulation of lipids within hepatocytes (1, 2). Its global prevalence has surged in recent years, establishing it as a leading cause of chronic liver diseases (3). A substantial body of research has demonstrated that NAFLD is intricately linked to metabolic-related conditions, including cardiovascular diseases and chronic kidney diseases, and significantly elevates the risk of developing type 2 diabetes mellitus (T2DM) (4, 5). Insulin resistance (IR) is a critical factor in the onset and progression of NAFLD and is acknowledged as a fundamental mechanism driving the transition from NAFLD to T2DM (6). The identification of simple and effective indicators for assessing the future risk of T2DM in patients with NAFLD is of paramount importance for the early screening and intervention of the disease. Recent studies have increasingly emphasized the heterogeneity in the pathophysiological links between NAFLD, insulin resistance, and cardiovascular risk. Variability in genetic backgrounds, hepatic fat content distribution, metabolic flexibility, and inflammatory responses contributes to this heterogeneity, which can affect disease progression and treatment responses (7–9). For instance, some individuals with NAFLD exhibit preserved insulin sensitivity and minimal cardiovascular risk, while others experience rapid metabolic deterioration despite similar levels of hepatic steatosis. These findings suggest that understanding such heterogeneity is essential for developing more targeted diagnostic and therapeutic strategies in NAFLD management. Conventional diabetes risk predictors (e.g., fasting glucose) may fail to capture this complexity, whereas TyG and TyG/HDL-c, as integrative indicators of glucose-lipid metabolism, could provide enhanced stratification in NAFLD. Therefore, this study specifically targets NAFLD patients to address unmet needs in precision risk assessment.

In recent years, the triglyceride-glucose index (TyG) has gained widespread application in assessing the risk of insulin resistance and associated metabolic disorders, owing to its high feasibility and predictive accuracy (10, 11). TyG, derived from fasting plasma glucose (FPG) and fasting triglycerides (TG), serves as an effective indicator of the body’s insulin sensitivity (12). Empirical studies have established a significant correlation between TyG and the risk of T2DM in both the general population and specific disease cohorts, underscoring its robust predictive value (13). However, insulin resistance is frequently accompanied by dyslipidemia, and HDL-c, a key anti-atherosclerotic lipoprotein, is intimately linked to insulin sensitivity (14). Consequently, the ratio of TyG to HDL-c (TyG/HDL-c) has been proposed as a novel metabolic risk assessment metric, potentially offering a more comprehensive reflection of the interplay between glucose and lipid metabolism disorders in the pathogenesis of T2DM than TyG alone (15, 16). Despite this, research on the predictive efficacy of TyG and TyG/HDL-c for T2DM risk specifically within the NAFLD population remains sparse, and its clinical significance warrants further investigation.

While previous studies have preliminarily investigated the association between TyG and the risk of T2DM, the majority have relied solely on linear regression analysis, thereby overlooking the potential non-linear relationship between TyG, TyG/HDL-c, and T2DM risk (17, 18). In clinical research, the influence of various metabolic indicators frequently does not adhere to a simple linear progression but rather operates within specific thresholds, with their risk effects potentially varying across different ranges. Moreover, variables such as sex, age, and metabolic status may modulate individuals’ sensitivity to insulin resistance, consequently impacting the predictive value of TyG and TyG/HDL-c across diverse populations (19–21). Therefore, it is imperative to further explore the non-linear relationship between TyG, TyG/HDL-c, and T2DM risk, as well as to assess their applicability across different subgroups, to enhance diabetes risk assessment in patients with NAFLD.

This study employs a retrospective cohort design to investigate the association between TyG, TyG/HDL-c, and the prospective risk of T2DM in individuals with NAFLD. It also aims to determine the presence of non-linear correlations and specific thresholds. Furthermore, the study evaluates the predictive utility of TyG and TyG/HDL-c across various subpopulations, stratified by gender, age, and metabolic status, through subgroup analyses to elucidate their clinical relevance. To enhance the robustness of the data, multiple imputation techniques are used to address missing data (22), and SMOTE is applied to balance the dataset (23). The relationship between TyG, TyG/HDL-c, and T2DM risk is assessed using multivariate logistic regression analysis, while restricted cubic spline (RCS) analysis is employed to explore non-linear trends. Subgroup analyses are further conducted to examine variations in predictive performance across different populations. This study is anticipated to refine the risk assessment framework for T2DM in patients with NAFLD, thereby equipping clinicians with more efficient and practical predictive tools. Additionally, identifying key thresholds for TyG and TyG/HDL-c will aid in more accurately assessing the risk of T2DM, providing a scientific basis for individualized interventions. Moreover, through the analysis of different subgroups, the applicability of these indicators in diverse populations can be further clarified, offering strong support for the advancement of precision medicine in the future. This study aims to provide a new scientific basis for early prediction of diabetes risk in patients with NAFLD and offer theoretical support for clinical decision-making and the development of individualized prevention strategies.

## 2 Materials and methods

### 2.1 Data source

**Figure 1:**
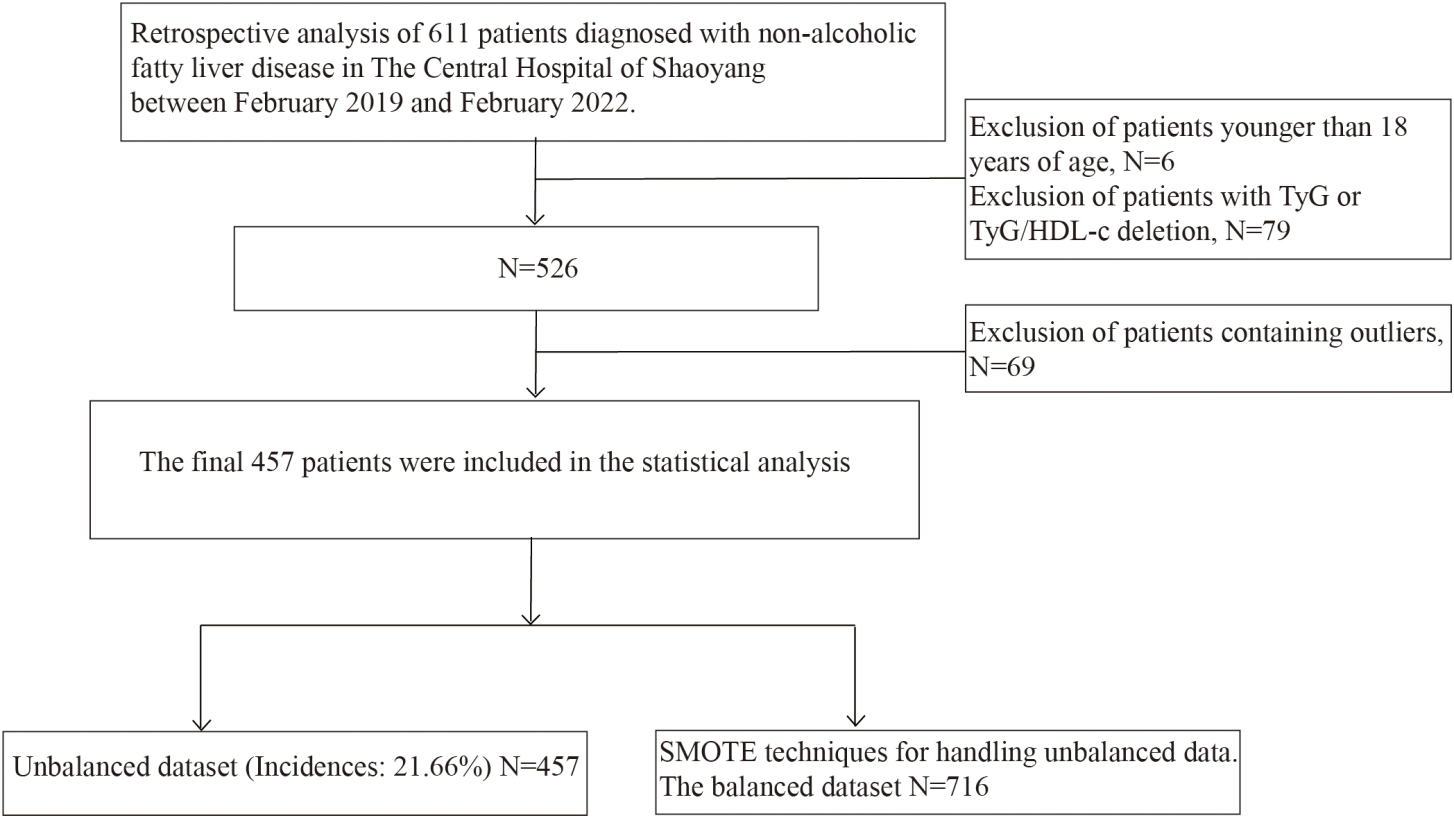
Flowchart of data processing.

This investigation utilized a retrospective cohort study design conducted at The Central Hospital of Shaoyang. The study initially enrolled 611 patients diagnosed with NAFLD at the hospital between February 2019 and February 2022. Participants were monitored over a three-year period to assess the incidence of diabetes. T2DM was diagnosed at baseline and during the follow-up period according to the 2023 American Diabetes Association (ADA) criteria (24), FPG levels were measured using the AU5800 automatic biochemical analyzer (Beckman Coulter, USA), and HbA1c was assessed using the VARIANT II analyzer (Bio-Rad, USA), based on high-performance liquid chromatography (HPLC). Medical records were also reviewed to identify new diabetes diagnoses and medication use. NAFLD diagnoses were confirmed via abdominal ultrasonography, and all participants were required to have a minimum of three years of follow-up data. The inclusion criteria were as follows: (1) initial diagnosis of NAFLD at The Central Hospital of Shaoyang, (2) absence of a diabetes diagnosis at baseline, and (3) availability of comprehensive clinical information and follow-up data. The exclusion criteria included: (1) age under 18 years, (2) presence of other chronic liver diseases (such as viral hepatitis, autoimmune liver disease, alcoholic liver disease, etc.), (3) presence of malignant tumors, pregnancy, or severe organ failure, (4) comorbid diabetes or use of hypoglycemic medications at baseline, and (5) loss to follow-up or incomplete critical data during the follow-up period. Ultimately, 457 eligible NAFLD patients were included in the analysis, of whom 99 (21.66%) developed diabetes over the three-year follow-up period. The detailed data screening process is illustrated in **Figure 1**. All data used in this study were approved by the ethics committee and met privacy protection requirements. As this was a retrospective study, the ethics committee approved our request for waiver of informed consent.

### 2.2 Study variables

#### 2.2.1 Exposure factors

The primary exposure factors in this study were TyG and TyG/HDL-c. The TyG index was calculated using the formula (25):


$$TyG=ln\left(\frac{FPG\;\left(mg/dL\right)\times TG\;\left(mg/dL\right)}{2}\right)$$

The TyG/HDL-c index was calculated using the formula (26):


$$TyG/HDL-c=\frac{TyG}{HDL-c\;\left(mg/dL\right)}$$

The determination of FPG, TG, and HDL-c was conducted using the Beckman Coulter AU5800 series fully automated biochemical analyzer.

#### 2.2.2 Covariates

To account for potential confounding variables, this study incorporated a comprehensive set of covariates, encompassing demographic characteristics, lifestyle factors, blood pressure indicators, underlying disease conditions, and laboratory test results. The demographic variables included age, sex, and marital status. Lifestyle factors were assessed through smoking and drinking status. Smoking status was categorized into three groups based on medical history records: non-smokers, ex-smokers, and smokers. Due to limitations in the electronic medical record system, alcohol consumption status could only be obtained as binary data (yes/no). Blood pressure indicators comprised systolic blood pressure (SBP) and diastolic blood pressure (DBP). Hypertension and coronary heart disease (CHD) were considered as underlying disease conditions. Furthermore, biochemical indicators such as total bilirubin (TBIL), alanine aminotransferase (ALT), aspartate aminotransferase (AST), urea, creatinine (CREA), and uric acid (UA) were collected to evaluate liver and kidney function. Blood routine indicators, including white blood cell count (WBC), red blood cell count (RBC), hemoglobin (HB), and platelet count (PLT), were also included. All covariates were obtained from patients’ electronic medical records and biochemical test results, with standardized measurements conducted in the clinical laboratory of The Central Hospital of Shaoyang.

#### 2.2.3 Outcome events

The primary outcome event of the study was the occurrence of T2DM, defined according to the 2023 standards of the American Diabetes Association (ADA). This included: (1) FPG ≥ 7.0 mmol/L (126 mg/dL); (2) Glycosylated hemoglobin (HbA1c) ≥ 6.5%; (3) Clinically diagnosed diabetes and taking hypoglycemic medications.

### 2.3 Ethics statement

This study is a retrospective analysis, and its design and methodology have been approved by the Medical Ethics Committee of The Central Hospital of Shaoyang (Ethics Approval Number: KY2025-002-04). Given the retrospective nature of this study and the exclusive use of anonymous clinical data that neither reveals patient privacy nor poses any direct risks to participants, the requirement for informed consent has been waived according to applicable ethical guidelines and regulations.

### 2.4 Statistical methods

The data analysis was executed utilizing R software (version 4.2.2) and Matlab software (version 2021a). Initially, a normality test was applied to continuous variables. Variables adhering to a normal distribution were reported as mean ± standard deviation (Mean ± SD) and inter-group comparisons were conducted using the t-test. Conversely, non-normally distributed data were presented as median (interquartile range) and analyzed via the Mann-Whitney U test. Categorical variables were described as frequencies (percentages) and compared using either the chi-square test or Fisher’s exact test. To address the issue of missing data, Multiple Imputation by Chained Equations (MICE) was employed to mitigate potential biases arising from missing values. Post-imputation, outlier detection and removal were conducted to ensure data integrity. To ensure data quality and minimize the impact of extreme values, outlier detection was performed using the Z-score method. For each continuous numerical variable, a standardized Z-value was calculated. Values with absolute Z-scores greater than 3 (|Z| > 3) were defined as outliers, and participants containing such outliers were excluded from the analysis, resulting in the removal of 69 cases. The quantity and distribution of these outliers across various variables are presented in **Supplementary Table S1**. After removing outliers, the SMOTE was implemented in Matlab 2021a to address the sample imbalance between groups with and without diabetes occurrence, thereby ensuring a balanced dataset for subsequent analyses. Prior to conducting the formal analysis, multicollinearity among all variables was evaluated using the Variance Inflation Factor (VIF), with a VIF greater than 10 indicating severe multicollinearity, which required adjustment. Additionally, PCA was employed to examine the distribution of variables in both the balanced and original datasets, assessing the impact of SMOTE on the data structure. The primary analytical approach in this study was multivariate logistic regression analysis, designed to evaluate the relationship between TyG, TyG/HDL-c, and the risk of diabetes in patients with NAFLD. Three regression models were developed: Model 1 assessed the crude association between TyG, TyG/HDL-c, and diabetes risk without adjusting for any covariates; Model 2 adjusted for age, sex, marital status, smoking, alcohol consumption, SBP, DBP, body mass index (BMI), hypertension, and CHD. In Model 3, adjustments were made for TBIL, ALT, AST, urea, CREA, UA, WBC, RBC, HB, and PLT, building upon Model 2, to enhance the control of confounding variables. A subgroup analysis was performed to assess the stability of TyG and TyG/HDL-c across different populations, stratified by variables such as sex, marital status, and pre-existing conditions. Interaction terms were incorporated to examine potential interactions between variables. Furthermore, RCS analysis was utilized to explore the nonlinear relationship between exposure factors and the risk of T2DM, identify threshold values, and evaluate P-nonlinear. A P-nonlinear value of less than 0.05 was considered indicative of a statistically significant nonlinear relationship. For optimal knot selection in the RCS, models with 3 to 7 knots were evaluated, and the model with the lowest Akaike Information Criterion (AIC) value was chosen as the final model. All analytical results were deemed statistically significant at a P-value of less than 0.05.

## 3 Results

### 3.1 Baseline characteristics of patients

Initially, our study enrolled 611 patients. Following the exclusion of six patients under the age of 18 and 79 patients with missing TyG or TyG/HDL-c values, the missing rates for each variable were calculated, as depicted in **Supplementary Figure S1**. A total of 74 participants (16.2%) were identified to have at least one missing value. The highest missing rates were observed for marital status, smoking, and drinking history (each 3.80%), while core clinical variables such as age, sex, liver function parameters, and outcome variables had no missing data. Missing values were subsequently estimated using multiple imputation techniques. After addressing outliers and excluding 69 patients with anomalous values, the final analysis included 457 patients diagnosed with nonalcoholic fatty liver disease, among whom 99 (21.66%) developed diabetes during the follow-up period. To address the class imbalance in the original dataset, the SMOTE was employed. A baseline characteristic analysis was conducted on both the balanced dataset (N = 716) and the unbalanced dataset (N = 457), comparing the clinical features between patients with nonalcoholic fatty liver disease who developed diabetes and those who did not. To ensure the robustness of the analysis, a multicollinearity assessment was performed on variables from both datasets. The results, presented in **Supplementary Table S2**, indicated that the VIF for all variables in both datasets was below 5, suggesting no significant multicollinearity issues and confirming the suitability of the variables for subsequent analyses.

In the balanced dataset (**Table 1**), no statistically significant differences were observed between the two groups concerning age, Sex, BMI, smoking, CHD, SBP, ALT, AST, urea, WBC, RBC, HB, and PLT. Nevertheless, the diabetes group exhibited a higher proportion of unmarried individuals (14.80% *vs*. 8.10%, P = 0.005), a lower proportion of alcohol consumption (4.75% *vs*. 8.66%, P = 0.036), a significantly elevated prevalence of hypertension (37.15% *vs*. 20.39%, P < 0.001), reduced DBP (80 ± 9 *vs*. 82 ± 11, P = 0.024), significantly lower levels of CREA (P < 0.001) and UA (P = 0.002), and significantly higher levels of TyG (P < 0.002) and TyG/HDL-c (P < 0.001). In the unbalanced dataset (**Supplementary Table S3**), the overall trends were consistent with those observed in the balanced dataset, demonstrating a significantly higher proportion of unmarried individuals in the diabetes group (16.16% *vs*. 8.10%, P = 0.017), a significant increase in the prevalence of hypertension (39.39% *vs*. 20.39%, P < 0.001), and significantly elevated levels of TyG (P < 0.001) and TyG/HDL-c (P < 0.001). Taken together, across both balanced and unbalanced datasets, the proportion of hypertension, unmarried status, and the indicators of TyG and TyG/HDL-c were significantly elevated in the NAFLD with diabetes group, suggesting that these factors may play important roles in the development and progression of NAFLD with diabetes.

**Table 1:** Baseline information table for balanced dataset.

Variables	Overall, N = 716^1^	NAFLD without DM, N = 358^1^	NAFLD with DM, N = 358^1^	p-value
Age	47 (37, 57)	48 (37, 57)	47 (37, 57)	0.917^2^
Sex				0.273^3^
Female	252 (35.20%)	133 (37.15%)	119 (33.24%)	
Male	464 (64.80%)	225 (62.85%)	239 (66.76%)	
BMI	26.2 (24.4, 28.7)	26.6 (24.5, 28.8)	26.0 (24.4, 28.3)	0.057^2^
Marital				0.005^3^
Married	634 (88.55%)	329 (91.90%)	305 (85.20%)	
Unmarried	82 (11.45%)	29 (8.10%)	53 (14.80%)	
Smoking				0.766^3^
Ex-smoker	131 (18.30%)	62 (17.32%)	69 (19.27%)	
Non-smoker	514 (71.79%)	259 (72.35%)	255 (71.23%)	
Smoker	71 (9.92%)	37 (10.34%)	34 (9.50%)	
Drinking				0.036^3^
No	668 (93.30%)	327 (91.34%)	341 (95.25%)	
Yes	48 (6.70%)	31 (8.66%)	17 (4.75%)	
Hypertension				<0.001^3^
No	510 (71.23%)	285 (79.61%)	225 (62.85%)	
Yes	206 (28.77%)	73 (20.39%)	133 (37.15%)	
CHD				0.469^3^
No	684 (95.53%)	340 (94.97%)	344 (96.09%)	
Yes	32 (4.47%)	18 (5.03%)	14 (3.91%)	
SBP	131 (121, 141)	130 (120, 142)	131 (123, 140)	0.856^2^
DBP	81 (74, 88)	82 (75, 89)	79 (74, 86)	0.021^2^
TBIL	14 (11, 18)	14 (10, 19)	14 (11, 18)	0.564^2^
ALT	30 (19, 48)	30 (19, 48)	30 (19, 48)	0.676^2^
AST	26 (20, 34)	25 (20, 35)	27 (20, 33)	0.809^2^
Urea	4.73 (3.80, 5.91)	4.84 (3.71, 6.00)	4.65 (3.84, 5.66)	0.520^2^
CREA	64 (53, 80)	68 (57, 85)	60 (49, 75)	<0.001^2^
UA	332 (277, 408)	349 (287, 426)	323 (269, 397)	0.005^2^
WBC	8.4 (6.5, 11.6)	7.8 (6.3, 11.0)	9.0 (6.6, 12.0)	0.010^2^
RBC	4.79 (4.41, 5.10)	4.79 (4.36, 5.18)	4.80 (4.46, 5.06)	0.796^2^
HB	142 ± 20	141 ± 19	142 ± 21	0.435^4^
PLT	232 (196, 270)	232 (197, 274)	232 (195, 269)	0.950^2^
TyG	2.38 (1.62, 3.86)	1.99 (1.30, 3.03)	2.94 (2.10, 4.51)	<0.001^2^
TyG/HDL-c	2.14 (1.28, 3.17)	1.72 (1.04, 2.61)	2.56 (1.76, 3.69)	<0.001^2^

^1^Median (Q1, Q3), n (%), Mean ± SD;

^2^Wilcoxon rank sum test;

^3^Pearson’s Chi-squared test;

^4^Welch Two Sample t-test.

### 3.2 Results of principal component analysis

In this study, PCA was utilized to assess the impact of the SMOTE on the distribution of variables within the dataset. Principal component loadings were calculated for both the unbalanced and balanced datasets, and their variations were compared to evaluate the effect of SMOTE on the data structure. The findings, presented in **Supplementary Table S4**, reveal that in the unbalanced dataset, the first principal component (PC1) was predominantly influenced by variables such as TyG (0.7909), HB (0.7407), TyG/HDL-c (0.6937), and RBC (0.6479). In the balanced dataset, the primary contributing variables for PC1 remained largely consistent (TyG: 0.7931, HB: 0.7499, TyG/HDL-c: 0.6813, RBC: 0.6566), indicating that the influence of these variables did not experience substantial changes following SMOTE processing. Furthermore, the contributions of WBC, ALT, and AST to PC2 were largely unchanged, suggesting that the SMOTE method did not significantly alter the principal component distribution of these variables. In the PC3 dimension, CREA, Urea, and UA exhibited higher loadings in the unbalanced dataset (CREA: 0.8156, Urea: 0.7355, UA: 0.5847). However, in the balanced dataset, the loading for CREA increased markedly to 0.9596, whereas the loadings for urea and UA exhibited only minor changes (urea: 0.7250, UA: 0.7244). This variation suggests that the application of the SMOTE may have influenced the distribution of renal function-related variables within this principal component dimension. Additionally, SBP and DBP contributed to the second principal component (PC2) in the balanced dataset (SBP: -0.3971, DBP: 0.4565), while the loading of PLT in the third principal component (PC3) decreased from 0.9664 in the unbalanced dataset to 0.6311. This indicates that the SMOTE method may have adjusted the principal component distribution of certain blood pressure and platelet-related variables. Overall, the PCA results indicate that SMOTE processing did not substantially alter the contribution patterns of most variables to the principal components, and the overall data structure remained stable. Only minor adjustments were observed in specific variables, such as CREA and PLT. This suggests that while SMOTE effectively balances the dataset, it exerts a limited impact on the global structure of the data, thereby enhancing the stability and reliability of subsequent modeling efforts.

### 3.3 Association between TyG, TyG/HDL-c, and the risk of diabetes in patients with NAFLD

In the balanced dataset, both TyG and TyG/HDL-c exhibited a significant dose-response relationship with the risk of diabetes among patients with NAFLD (**Table 2**). For instance, when TyG was analyzed as a continuous variable, its unadjusted OR was 1.66 (95% CI: 1.48-1.87). Upon multivariable adjustment, the effect size progressively increased, reaching an OR of 1.96 (95% CI: 1.67-2.30) in Model 3. Following standardization, the OR for Model 3 further escalated to 2.63 (95% CI: 2.09-3.31). Quartile analysis revealed that the risk of diabetes in the highest quartile (Q4) was 12.93 times greater than that in the lowest quartile (Q1) (95% CI: 6.94-24.12), with a significant dose-response trend (P for trend < 0.001). TyG/HDL-c demonstrated a comparable pattern, with an OR of 1.90 (95% CI: 1.60-2.26) as a continuous variable in Model 3, which increased to 2.47 (95% CI: 1.94-3.14) following standardization. The risk in the Q4 group was elevated by 7.57 times compared to the Q1 group (95% CI: 4.34-13.22).

**Table 2:** Association between TyG, TyG/HDL-c and the risk of diabetes in patients with NAFLD in the balanced dataset.

Variables	Model 1		Model 2		Model 3	
	OR (95% CI)	P	OR (95% CI)	P	OR (95% CI)	P
TyG (continuous)	1.66 (1.48-1.87)	<0.001	1.79 (1.56-2.05)	<0.001	1.96 (1.67-2.30)	<0.001
TyG (standardized)	2.08 (1.76-2.46)	<0.001	2.31 (1.90-2.81)	<0.001	2.63 (2.09-3.31)	<0.001
TyG						
Q1						
Q2	2.61 (1.67-4.07)	<0.001	3.39 (2.10-5.46)	<0.001	3.72 (2.26-6.12)	<0.001
Q3	3.25 (2.08-5.08)	<0.001	3.73 (2.30-6.07)	<0.001	4.55 (2.69-7.67)	<0.001
Q4	7.56 (4.72-12.09)	<0.001	10.51 (6.09-18.14)	<0.001	12.93 (6.94-24.12)	<0.001
P for trend		<0.001		<0.001		<0.001
TyG/HDL-c (continuous)	1.74 (1.52-1.99)	<0.001	1.87 (1.60-2.19)	<0.001	1.90 (1.60-2.26)	<0.001
TyG/HDL-c (standardized)	2.18 (1.80-2.64)	<0.001	2.41 (1.93-3.01)	<0.001	2.47 (1.94-3.14)	<0.001
TyG/HDL-c						
Q1						
Q2	1.79 (1.16-2.78)	0.009	1.85 (1.17-2.92)	0.009	1.84 (1.14-2.98)	0.013
Q3	3.08 (1.99-4.76)	<0.001	3.83 (2.38-6.17)	<0.001	4.25 (2.56-7.07)	<0.001
Q4	5.81 (3.68-9.15)	<0.001	6.98 (4.16-11.70)	<0.001	7.57 (4.34-13.22)	<0.001
P for trend		<0.001		<0.001		<0.001

^1^OR, Odds Ratio, CI, Confidence Interval; Model 1: no covariates were adjusted; Model 2: adjusted for Age, Sex, Marital, Smoking, Drinking, SBP, DBP, BMI, Hypertension, and CHD; Model 3: adjusted for Age, Sex, Marital, Smoking, Drinking, SBP, DBP, BMI, Hypertension, CHD, TBIL, ALT, AST, Urea, CREA, UA, WBC, RBC, HB, and PLT.

In the unbalanced dataset, the fundamental trends remained consistent, albeit with slightly diminished effect sizes. The OR for TyG as a continuous variable in Model 3 was 1.90 (95% CI: 1.52-2.39), increasing to 2.44 (95% CI: 1.78-3.34) after standardization. In the fourth quartile (Q4) group, the risk was 6.37 times greater than that observed in the first quartile (Q1) group (95% CI: 2.68-15.16). Regarding TyG/HDL-c, the OR as a continuous variable in Model 3 was 1.68 (95% CI: 1.34-2.10), which increased to 2.09 (95% CI: 1.52-2.87) following standardization. The risk in the Q4 group was elevated by 5.67 times relative to the Q1 group (95% CI: 2.45-13.12). Although the overall trends observed in the unbalanced dataset were consistent with those in the balanced dataset, certain subgroups did not achieve statistical significance. As detailed in **Supplementary Table S5**, when TyG was analyzed as a continuous variable, the unadjusted OR in Model 1 was 1.60 (95% CI: 1.36-1.89, P < 0.001), which increased to 1.76 (95% CI: 1.45-2.14, P < 0.001) and 1.90 (95% CI: 1.52-2.39, P < 0.001) in Models 2 and 3, respectively. The standardized TyG variable exhibited an OR of 2.44 (95% CI: 1.78-3.34, P < 0.001) in Model 3. In the quartile analysis, the Q4 group demonstrated the highest risk of diabetes in Model 3 (OR = 6.37, 95% CI: 2.68-15.16, P < 0.001), whereas the Q2 group did not exhibit a statistically significant difference (P > 0.05). The analysis results from the unbalanced dataset also corroborated the positive association between TyG/HDL-c and diabetes risk. In Model 3, the OR for TyG/HDL-c as a continuous variable was 1.68 (95% CI: 1.34-2.10, P < 0.001), rising to 2.09 (95% CI: 1.52-2.87, P < 0.001) after standardization. Quartile analysis revealed an OR of 5.67 for the Q4 group (95% CI: 2.45-13.12, P < 0.001).

### 3.4 Subgroup analysis results

**Figure 2:**
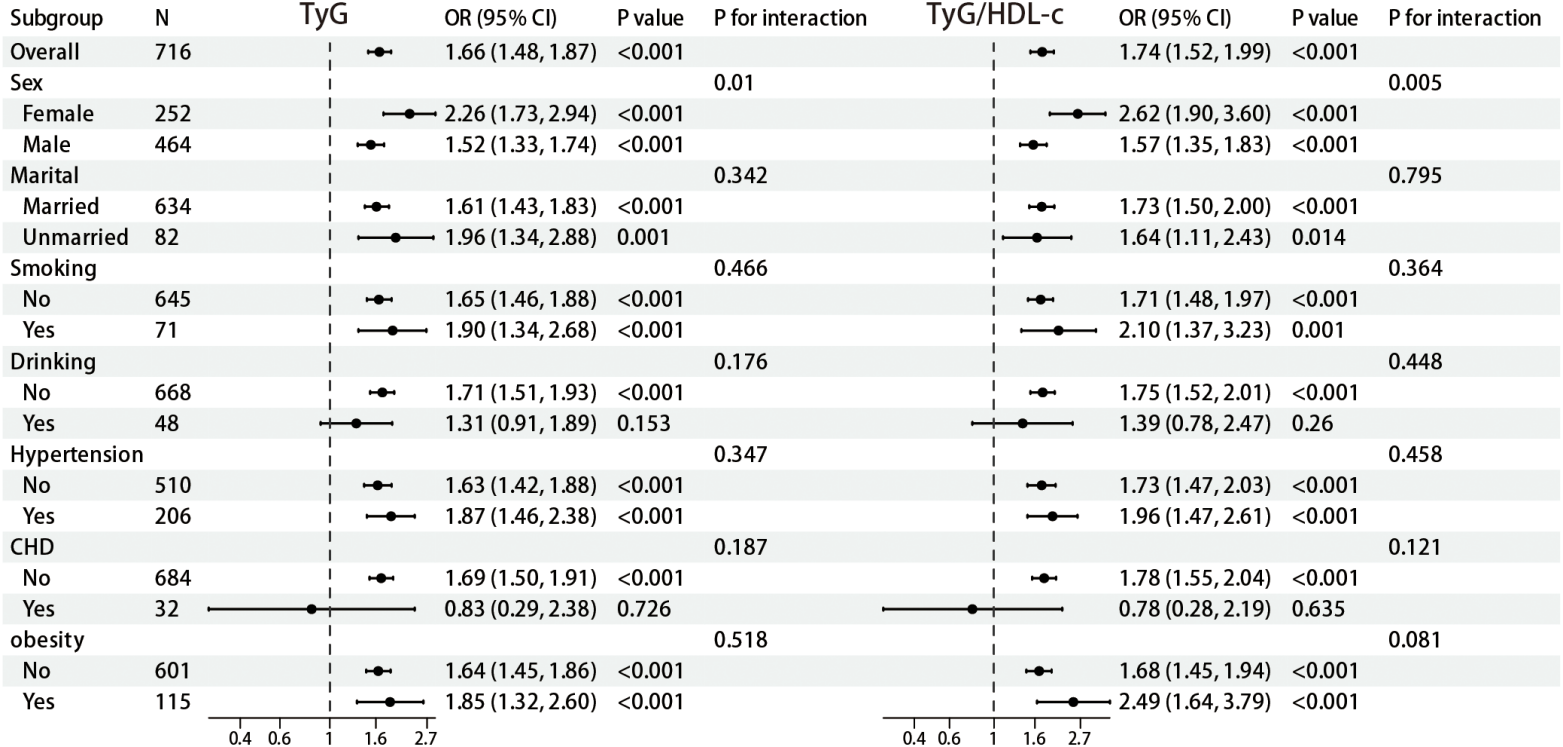
Forest map for subgroup analysis.

To further elucidate the relationship between TyG, TyG/HDL-c, and diabetes risk among patients with NAFLD, we performed stratified analyses across various subgroups within a balanced dataset. The findings are illustrated in **Figure 2**. In the overall cohort, TyG demonstrated a significant positive association with diabetes risk (OR = 1.66, 95% CI: 1.48-1.87, P < 0.001). Gender-stratified analysis indicated a more pronounced association in females compared to males (females: OR = 2.26, 95% CI: 1.73-2.94, P < 0.001; males: OR = 1.52, 95% CI: 1.33-1.74, P < 0.001; P-interaction = 0.01), suggesting a potential gender-modulating effect on this relationship. Analyses of subgroups defined by marital status, smoking habits, alcohol consumption, hypertension, and CHD also revealed significant associations between TyG and diabetes risk (P < 0.001); however, interaction analyses did not identify statistically significant interactions (P > 0.05). Subgroup analysis stratified by obesity status (BMI ≥ 30.0 kg/m²) demonstrated that obese individuals exhibited a stronger association between diabetes risk in NAFLD patients (OR = 1.85, 95% CI: 1.32-2.60, P < 0.001) compared with non-obese individuals (OR = 1.64, 95% CI: 1.45-1.86, P < 0.001), however, no statistically significant interaction was observed (P-interaction = 0.518). Similarly, TyG/HDL-c was significantly positively associated with diabetes risk in the overall population (OR = 1.74, 95% CI: 1.52-1.99, P < 0.001). In the gender-stratified analysis, the impact of TyG/HDL-c on diabetes risk was stronger in females than in males (females OR = 2.62, 95% CI: 1.90-3.60, P < 0.001; males OR = 1.57, 95% CI: 1.35-1.83, P < 0.001; P-interaction = 0.005), further supporting the potential influence of gender on this relationship. Additionally, analysis results from subgroups based on marital status, smoking, alcohol consumption, hypertension, and CHD were consistent with the overall trend, indicating that TyG/HDL-c is an important predictor of diabetes risk (P < 0.05), although the interactions did not reach statistical significance (P > 0.05). In obese individuals, the association was notably stronger (OR = 2.49, 95% CI: 1.64–3.79, P < 0.001) than in non-obese individuals (OR = 1.68, 95% CI: 1.45–1.94, P < 0.001), though the interaction did not reach statistical significance (P-interaction = 0.081).

### 3.5 The nonlinear relationship between TyG, TyG/HDL-c, and the risk of diabetes in patients with NAFLD

**Figure 3:**
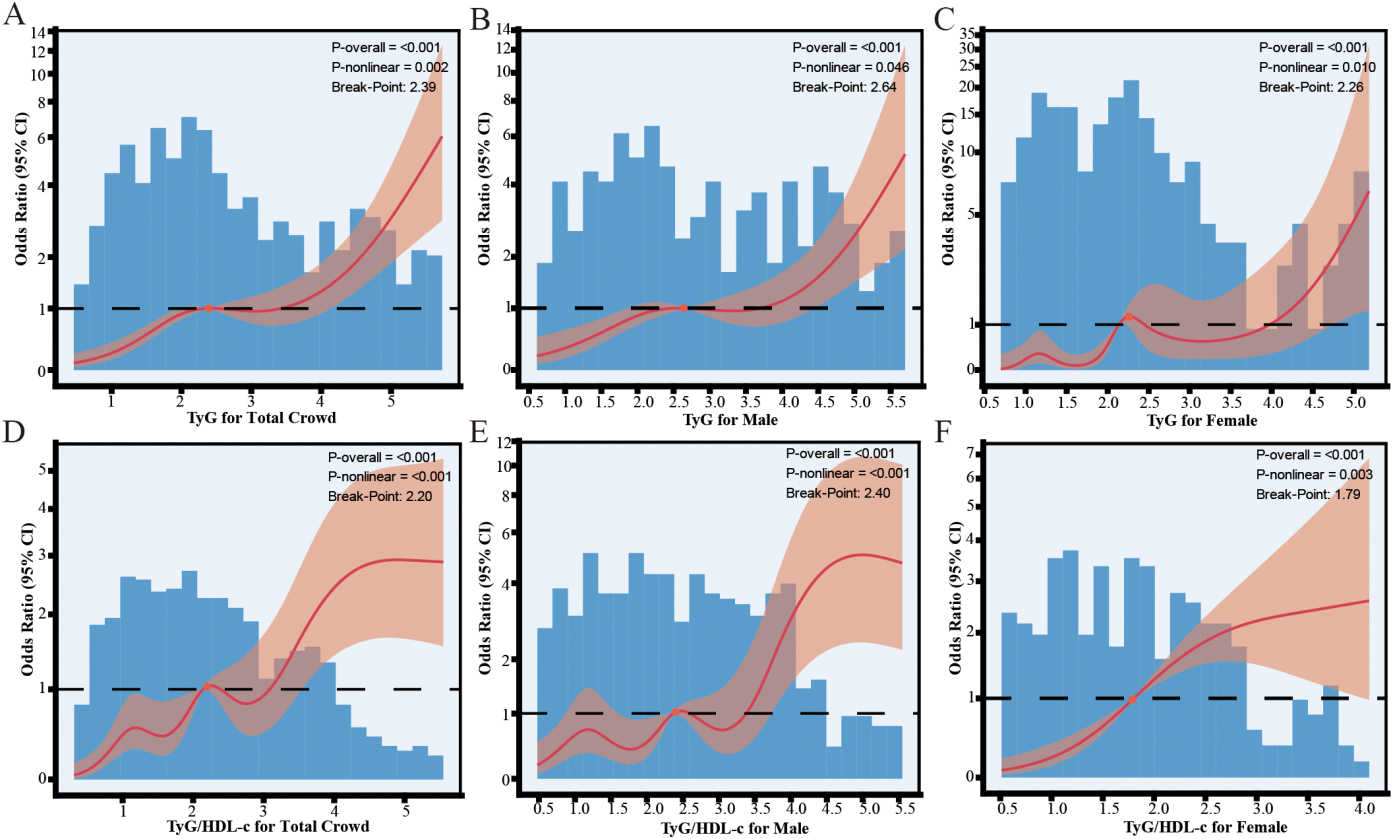
Nonlinear relationship between TyG, TyG/HDL-c, and the Risk of Diabetes in Patients with NAFLD. **(A)** TyG for total crowd; **(B)** TyG for male; **(C)** TyG for female; **(D)** TyG/HDL-c for total crowd; **(E)** TyG/HDL-c for male; **(F)** TyG/HDL-c for female.

In the overall population, the analysis using a restricted cubic spline curve (**Figure 3A**) demonstrated a nonlinear association between TyG values and outcome events (P-nonlinear < 0.001). As TyG values increased from 0 to 2.39, the OR value progressively rose, indicating a positive correlation between elevated TyG levels and an increased risk of outcome events. However, within the range of TyG values from approximately 2.40 to 3.20, the rate of increase in the curve began to decelerate, suggesting a reduced rate of risk escalation. Beyond a TyG value of 3.20, the curve continued to ascend, signifying an enhanced impact of TyG on outcome events. In the male cohort (**Figure 3B**), the relationship between TyG and outcome events similarly exhibited a nonlinear pattern (P-nonlinear = 0.046). With rising TyG values, the OR value consistently increased, with an inflection point occurring at a TyG of approximately 2.64, where the rate of increase slightly diminished, yet the risk continued to escalate thereafter. In the female cohort (**Figure 3C**), the RCS analysis revealed a multiphasic alteration in the relationship between TyG and outcome events, characterized by a local peak at a TyG of approximately 1.20, followed by a slight reduction in the OR. However, the risk rose again after TyG 1.7, particularly above TyG 3.0, where the odds continued to increase. This result suggests that the impact of TyG on outcome events in the female population may be modulated by other factors, exhibiting a more complex risk pattern.

In the overall population, as depicted in **Figure 3D**, a significant nonlinear association was observed between TyG/HDL-c values and outcome events (P-nonlinear < 0.001). As TyG/HDL-c values increased, there was a general upward trend in risk, with an inflection point identified between 1.2 and 2.2, suggesting a more intricate variation in risk within this range. Beyond a value of 2.8, the OR exhibited a relatively stable upward trend, further corroborating the nonlinear influence of TyG/HDL-c on outcome events. In the male cohort, shown in **Figure 3E**, the relationship between TyG/HDL-c and outcome events also exhibited nonlinear characteristics. Around a TyG/HDL-c value of 2.4, the curve displayed slight fluctuations before stabilizing; however, when the value surpassed 3.3, the risk increased markedly. In the female cohort, as illustrated in **Figure 3F**, the risk associated with TyG/HDL-c initially demonstrated a rapid increase followed by a slower upward trend. At approximately 1.79, the OR value increased significantly. Although the risk continued to rise thereafter, the rate of increase decelerated, particularly as it approached a value of 3.0. This suggests that the impact of TyG/HDL-c on outcome events in the female population may reach a saturation point at higher levels.

### 3.6 Analysis of the ROC curve

**Figure 4:**
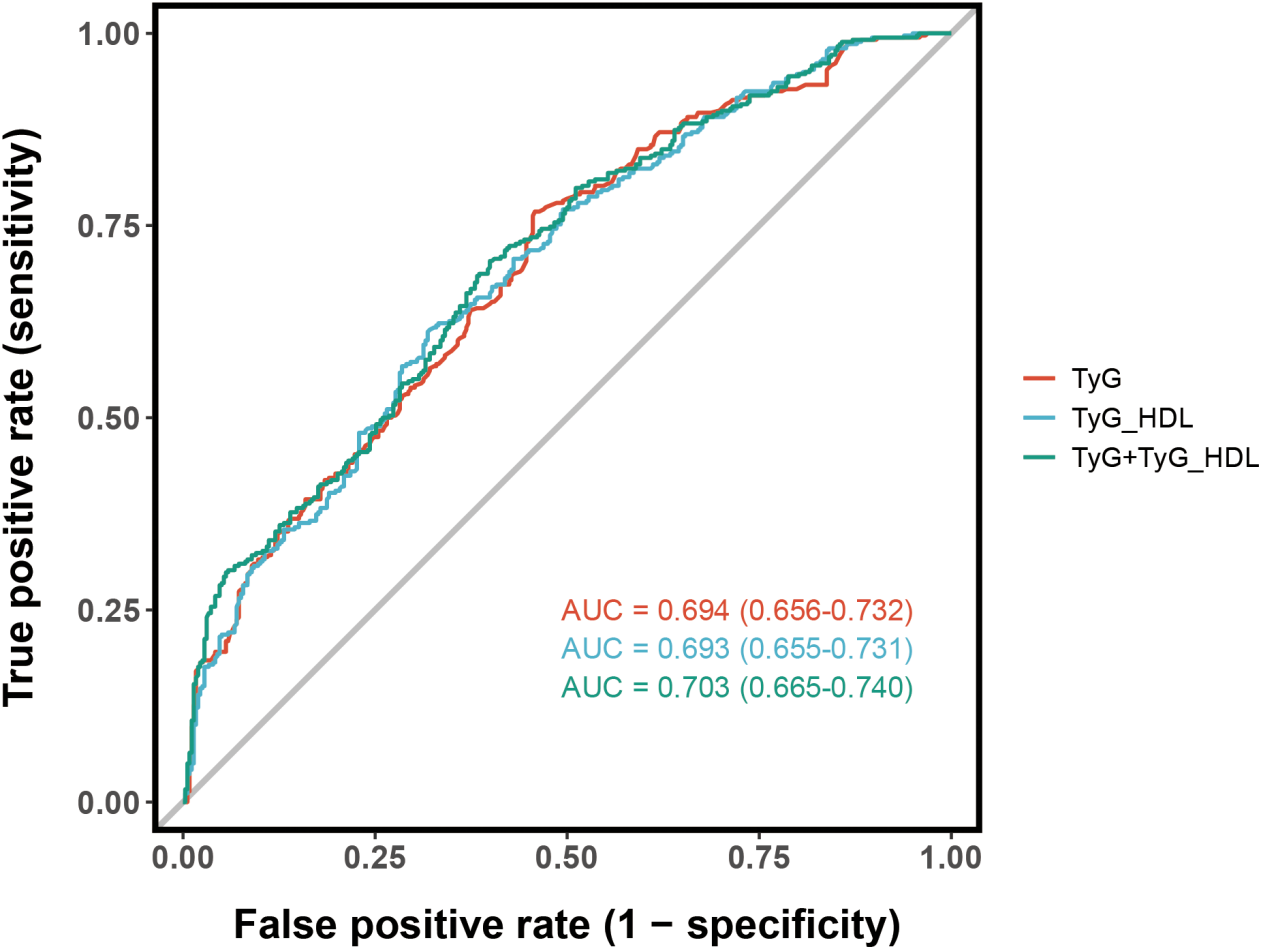
Analysis of the ROC curve.

In this research, ROC curve analysis was utilized to assess the ability of TyG and TyG/HDL-c to distinguish outcome events. As shown in **Figure 4**, the AUC for TyG alone was 0.694 (95% CI: 0.656-0.732). For TyG/HDL-c, the AUC was 0.693 (95% CI: 0.655-0.731), indicating similar predictive capabilities. When TyG and TyG/HDL-c were combined, the AUC rose to 0.703 (95% CI: 0.665-0.740), suggesting enhanced predictive performance. These results show that TyG and TyG/HDL-c can predict diabetes risk in NAFLD patients, and their combination may improve prediction.

## 4 Discussion

Utilizing a retrospective cohort from The Central Hospital of Shaoyang, this study conducted a systematic analysis of the association between TyG, its derivative index TyG/HDL-c, and the risk of diabetes mellitus in patients with NAFLD. The findings revealed that both TyG and TyG/HDL-c significantly elevated the prospective risk of DM in individuals with NAFLD, demonstrating a nonlinear relationship. Additionally, subgroup analysis by gender indicated variations in the predictive value of these indices between males and females. These results suggest that TyG and its derivative index, TyG/HDL-c, are significantly associated with the future risk of diabetes in patients with NAFLD and may provide **Supplementary Information** for risk stratification in clinical practice. Furthermore, their associations with diabetes risk may reflect underlying metabolic disturbances that warrant further investigation.

The study identified a significant positive correlation between TyG and the risk of DM in NAFLD patients (OR = 1.96, 95% CI: 1.67-2.30, P < 0.001). This implies that TyG is not solely a metabolic marker of insulin resistance but may also contribute directly to the pathogenesis of DM (27). Previous research has demonstrated a strong association between elevated TyG levels and disruptions in insulin signaling pathways, notably the inhibition of the IRS-1/PI3K/Akt pathway (28). This disruption results in diminished insulin-mediated glucose uptake and contributes to the development of insulin resistance. Furthermore, elevated TyG levels are frequently linked to chronic inflammatory responses, characterized by increased concentrations of proinflammatory cytokines such as TNF-α and IL-6, which may further impede insulin receptor signaling and exacerbate insulin resistance (29, 30).

Recent studies have also suggested that TyG may have potential predictive value beyond diabetes, such as in cancer development. For example, in bladder cancer—a common malignancy in elderly populations—TyG was shown to be elevated and significantly associated with insulin resistance and NAFLD, implying a potential link between metabolic dysfunction and tumorigenesis (31). This is supported by evidence from a cross-sectional study demonstrating increased NAFLD prevalence in non-metastatic bladder cancer patients and its correlation with TyG levels. Furthermore, diabetes itself has been identified as a risk factor for bladder cancer, as shown by meta-analyses demonstrating a statistically significant increased incidence of bladder cancer in individuals with diabetes compared to those without (32). These findings collectively suggest that TyG may serve not only as a marker of insulin resistance but also as an indicator of broader metabolic and oncological risks, especially in NAFLD populations.

Conversely, TyG/HDL-c has been shown to significantly elevate the risk of developing diabetes mellitus (OR=1.90, 95% CI: 1.60-2.26, P<0.001). HDL-c is known for its antioxidant (33), anti-inflammatory (34, 35), and cholesterol reverse transport-promoting properties (36), and it plays a crucial role in preserving insulin sensitivity (37). Reduced HDL-c levels may diminish its protective effects on insulin signaling pathways, thereby worsening insulin resistance (38). Additionally, low HDL-c levels may enhance adipose tissue inflammation and lipotoxicity by affecting the PPAR-γ signaling pathway, thus accelerating the progression of insulin resistance (39). Therefore, TyG/HDL-c may provide a more comprehensive reflection of the combined effects of insulin resistance and lipid metabolism abnormalities than TyG alone.

This study conducted an in-depth examination of the nonlinear relationship between TyG, TyG/HDL-c, and diabetes risk through the application of RCS analysis. The findings revealed a significant increase in diabetes risk when TyG surpassed 2.39 (p-nonlinear=0.002), whereas the inflection point for TyG/HDL-c was identified at 2.20 (p-nonlinear<0.001). This nonlinear relationship indicates that once metabolic disorders reach a specific threshold, the exacerbation of insulin resistance may initiate a series of reactions, including β-cell dysfunction (40), increased glucose toxicity (41), and abnormal glucagon secretion (42), thereby expediting the onset of diabetes. The identification of this threshold effect holds substantial implications for clinical intervention strategies, underscoring the importance of intensified metabolic management in patients with TyG or TyG/HDL-c values nearing these thresholds to avert rapid diabetes progression.

The subgroup analysis demonstrated a significant influence of gender on the predictive efficacy of TyG and TyG/HDL-c, as indicated by a P-value for interaction of less than 0.05. This observation may be explained by gender-specific endocrine regulatory mechanisms (43). Prior to menopause, women typically exhibit elevated estrogen levels, which can enhance insulin sensitivity by activating the AMPK signaling pathway and reducing visceral fat accumulation, thereby decreasing the risk of insulin resistance (44, 45). Conversely, the postmenopausal decline in estrogen levels may result in diminished insulin sensitivity, increasing the susceptibility of women to diabetes (46, 47). Furthermore, men generally present a higher risk of abdominal obesity, which is associated with gender-specific differences in testosterone levels, adipose tissue inflammation, and adiponectin levels (48). Consequently, integrating gender-specific metabolic characteristics into diabetes risk assessment models may improve their clinical relevance and applicability.

With regard to lifestyle factors, this study classified smoking status into three categories: non-smoker, ex-smoker, and smoker, allowing for a more precise adjustment of smoking-related confounding effects. However, data on alcohol consumption were limited to a binary classification (yes *vs*. no) due to the restrictions of the electronic medical record system. Despite this limitation, the influence of alcohol on triglyceride metabolism cannot be ignored. Moderate alcohol intake has been shown to elevate plasma triglyceride levels and influence metabolic markers such as TyG (49). Furthermore, evidence suggests that even moderate alcohol consumption may accelerate fibrosis progression in NAFLD and exert a synergistic effect with type 2 diabetes mellitus, further complicating disease outcomes (50).

In addition, although NAFLD is traditionally considered non-alcohol-related, growing evidence indicates that NAFLD and alcoholic fatty liver disease (AFLD) share many common pathogenic pathways. These include oxidative stress, endoplasmic reticulum stress, mitochondrial dysfunction, and activation of inflammatory signaling cascades. These overlapping mechanisms suggest a convergent pathway of liver injury regardless of alcohol involvement, and highlight the need to consider these similarities in both mechanistic research and clinical management strategies (51).

Although the predictive performance of TyG and TyG/HDL-c as independent biomarkers for T2DM is relatively modest, their clinical utility may lie within a broader framework of metabolic health risk assessment. As highlighted in a recent review by Stefan and Schulze (2023), the current scientific focus is shifting toward cardiometabolic risk clustering and metabolic health stratification, aiming to better characterize subpopulations with distinct risk profiles (52). In this context, TyG and its derivatives, by reflecting hepatic lipid accumulation and insulin resistance, may serve as convenient and cost-effective markers that complement existing models. Their incorporation into cardiometabolic clustering algorithms could facilitate the identification of high-risk populations, particularly within specific BMI strata or among patients with NAFLD, thereby enhancing the precision of diabetes and CVD prevention and management strategies.

The present study demonstrates several strengths: (1) It utilized a relatively large cohort of patients with NAFLD and conducted a 3-year follow-up, thereby enhancing the reliability of the findings. (2) The study employed multiple imputation methods to address missing data and applied the SMOTE to balance the dataset, thereby strengthening the model’s robustness. (3) Restricted cubic spline analysis was used to investigate the nonlinear relationships of TyG and TyG/HDL-c, elucidating their potential threshold effects. Nonetheless, several limitations must be acknowledged. Firstly, as a single-center retrospective study, there is a possibility of selection bias, which necessitates further validation through multi-center prospective studies. Secondly, despite adjustments for various confounding factors, the potential influence of unmeasured variables on the results cannot be entirely excluded. Furthermore, the study did not explore the relationships between TyG and TyG/HDL-c with other metabolic syndrome-related indicators (such as insulin and homeostasis model assessment of insulin resistance [HOMA-IR]), which presents an opportunity for future research.

## 5 Conclusion

This study substantiates that both TyG and TyG/HDL-c are significantly correlated with the risk of diabetes onset in individuals with NAFLD, demonstrating nonlinear associations. Additionally, the predictive efficacy of these indices may be modulated by gender, indicating the necessity of incorporating gender considerations into diabetes risk assessment protocols in clinical settings. Future mechanistic investigations are warranted to elucidate the precise roles of TyG and TyG/HDL-c in insulin signaling pathways, inflammatory processes, and the regulation of lipid metabolism. Such insights could enhance early screening and intervention strategies aimed at mitigating diabetes risk among NAFLD patients.

## Data Availability

The raw data supporting the conclusions of this article will be made available by the authors, without undue reservation.

## Supplementary material

Supplementary Figure 1 Lollipop chart of missing rates for study variables.
