# Supplementary figures and images for: Study on the correlation between triglyceride glucose index, triglyceride glucose index to high-density lipoprotein cholesterol ratio, and the risk of diabetes in nonalcoholic fatty liver disease
Source: Front Endocrinol (Lausanne). 2025 Jun 23;16:1594548. doi: 10.3389/fendo.2025.1594548 (PMC12229875; doi:10.3389/fendo.2025.1594548)

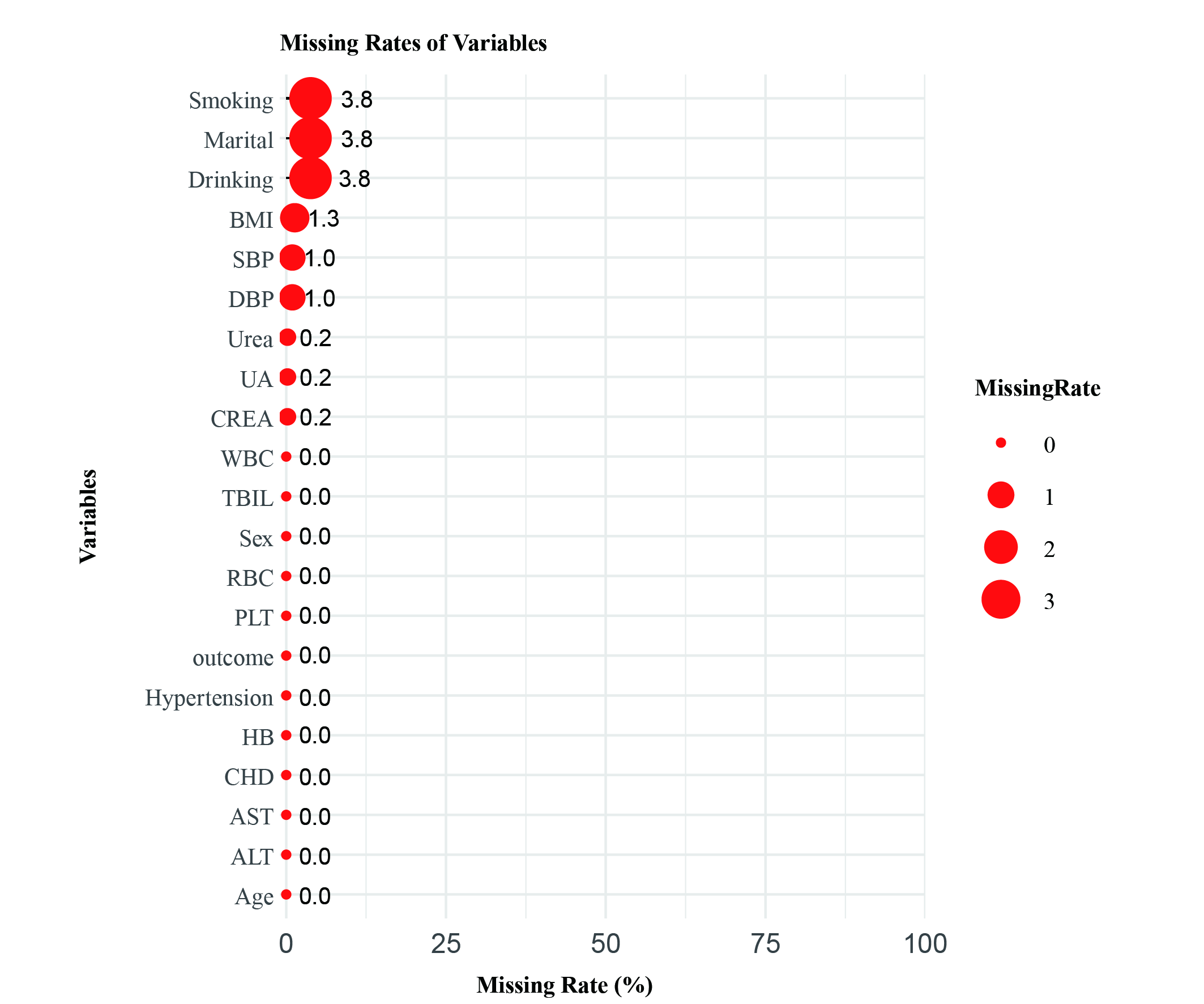

Supplement: Supplementary Figure 1 — Lollipop chart of missing rates for study variables. [file Image1.tif]
