# Supplementary material for: Study on the correlation between triglyceride glucose index, triglyceride glucose index to high-density lipoprotein cholesterol ratio, and the risk of diabetes in nonalcoholic fatty liver disease
Source: Front Endocrinol (Lausanne). 2025 Jun 23;16:1594548. doi: 10.3389/fendo.2025.1594548 (PMC12229875; doi:10.3389/fendo.2025.1594548)
Supplement: Supplementary file 4 [file Table3.docx]

Supplementary table 3. Baseline information table for unbalanced dataset.

| **Variables** | **Overall,**  **N = 457^1^** | **NAFLD without DM, N = 358^1^** | **NAFLD with DM, N = 99^1^** | **p-value** |
| --- | --- | --- | --- | --- |
| **Age** | 48 (36, 57) | 48 (37, 57) | 47 (35, 59) | 0.823^2^ |
| **Sex** |  |  |  | 0.208^3^ |
| Female | 163 (35.67%) | 133 (37.15%) | 30 (30.30%) |  |
| Male | 294 (64.33%) | 225 (62.85%) | 69 (69.70%) |  |
| **BMI** | 26.3 (24.4, 28.8) | 26.6 (24.5, 28.8) | 25.6 (24.0, 29.4) | 0.134^2^ |
| **Marital** |  |  |  | 0.017^3^ |
| Married | 412 (90.15%) | 329 (91.90%) | 83 (83.84%) |  |
| Unmarried | 45 (9.85%) | 29 (8.10%) | 16 (16.16%) |  |
| **Smoking** |  |  |  | 0.628^3^ |
| Ex-smoker | 83 (18.16%) | 62 (17.32%) | 21 (21.21%) |  |
| Non-smoker | 326 (71.33%) | 259 (72.35%) | 67 (67.68%) |  |
| Smoker | 48 (10.50%) | 37 (10.34%) | 11 (11.11%) |  |
| **Drinking** |  |  |  | 0.238^3^ |
| No | 421 (92.12%) | 327 (91.34%) | 94 (94.95%) |  |
| Yes | 36 (7.88%) | 31 (8.66%) | 5 (5.05%) |  |
| **Hypertension** |  |  |  | <0.001^3^ |
| No | 345 (75.49%) | 285 (79.61%) | 60 (60.61%) |  |
| Yes | 112 (24.51%) | 73 (20.39%) | 39 (39.39%) |  |
| **CHD** |  |  |  | 0.797^4^ |
| No | 435 (95.19%) | 340 (94.97%) | 95 (95.96%) |  |
| Yes | 22 (4.81%) | 18 (5.03%) | 4 (4.04%) |  |
| **SBP** | 131 ± 18 | 131 ± 18 | 131 ± 16 | 0.685^5^ |
| **DBP** | 81 (74, 88) | 82 (75, 89) | 79 (74, 87) | 0.105^2^ |
| **TBIL** | 14 (11, 19) | 14 (10, 19) | 15 (11, 18) | 0.849^2^ |
| **ALT** | 29 (19, 48) | 30 (19, 48) | 29 (18, 51) | 0.796^2^ |
| **AST** | 25 (20, 35) | 25 (20, 35) | 26 (19, 34) | 0.807^2^ |
| **Urea** | 4.81 (3.73, 6.00) | 4.84 (3.71, 6.00) | 4.68 (3.77, 5.77) | 0.823^2^ |
| **CREA** | 66 (55, 83) | 68 (57, 85) | 61 (46, 76) | <0.001^2^ |
| **UA** | 341 (279, 417) | 349 (287, 426) | 326 (261, 405) | 0.084^2^ |
| **WBC** | 8.0 (6.3, 11.2) | 7.8 (6.3, 11.0) | 8.4 (6.5, 13.0) | 0.208^2^ |
| **RBC** | 4.79 (4.37, 5.17) | 4.79 (4.36, 5.18) | 4.79 (4.44, 5.13) | 0.975^2^ |
| **HB** | 141 ± 20 | 141 ± 19 | 143 ± 23 | 0.505^5^ |
| **PLT** | 233 (195, 273) | 232 (197, 274) | 235 (193, 271) | 0.843^2^ |
| **TyG** | 2.15 (1.38, 3.34) | 1.99 (1.30, 3.03) | 2.79 (1.90, 4.60) | <0.001^2^ |
| **TyG/HDL-c** | 1.86 (1.12, 2.85) | 1.72 (1.04, 2.61) | 2.66 (1.61, 3.75) | <0.001^2^ |

^1^Mean ± SD, n(%); ^2^Welch Two Sample t-test; ^3^Pearson's Chi-squared test.
