# Supplementary material for: Study on the correlation between triglyceride glucose index, triglyceride glucose index to high-density lipoprotein cholesterol ratio, and the risk of diabetes in nonalcoholic fatty liver disease
Source: Front Endocrinol (Lausanne). 2025 Jun 23;16:1594548. doi: 10.3389/fendo.2025.1594548 (PMC12229875; doi:10.3389/fendo.2025.1594548)
Supplement: Supplementary file 5 [file Table4.docx]

Supplementary table 4. Principal component analysis component loadings plots for unbalanced and balanced datasets.

| variables | Unbalanced data set | | | | Balanced data set | | | |
| --- | --- | --- | --- | --- | --- | --- | --- | --- |
|  | pc1 | pc2 | pc3 | Uniqueness | pc1 | pc2 | pc3 | Uniqueness |
| TyG | 0.7909658 |  |  | 0.3471391 | 0.7931452 |  |  | 0.3298483 |
| HB | 0.7406886 |  |  | 0.4253911 | 0.7498774 |  |  | 0.4143419 |
| TyG/HDL-c | 0.6936907 |  |  | 0.4941901 | 0.6813407 |  |  | 0.5060687 |
| RBC | 0.6479055 |  |  | 0.4915669 | 0.6565956 |  |  | 0.4821424 |
| Age | -0.5633549 | -0.3339563 |  | 0.5697980 | -0.5549696 | -0.3578647 |  | 0.5570052 |
| WBC | 0.5054037 |  |  | 0.7436589 | 0.5296731 |  |  | 0.7154809 |
| ALT |  | 0.8528924 |  | 0.2698583 |  | 0.8541566 |  | 0.2689891 |
| AST |  | 0.8125664 |  | 0.3277542 |  | 0.8173342 |  | 0.3153309 |
| TBIL |  | 0.4762365 |  | 0.7613186 |  | 0.4885673 |  | 0.7511533 |
| BMI |  | 0.3578699 |  | 0.8405358 |  | 0.3707227 |  | 0.8037493 |
| CREA |  |  | 0.8156345 | 0.2841365 |  |  |  | 0.9595870 |
| Urea |  |  | 0.7355017 | 0.3759773 |  |  | 0.7250189 | 0.4558860 |
| UA |  |  | 0.5847230 | 0.5679309 |  |  | 0.7243883 | 0.4745086 |
| SBP |  |  |  | 0.8631257 | -0.3970883 |  | 0.5105818 | 0.5718274 |
| DBP |  |  |  | 0.8752579 |  |  | 0.4564911 | 0.7431029 |
| PLT |  |  |  | 0.9663627 | -0.3000678 | -0.3170956 | 0.4222845 | 0.6310855 |
