# Supplementary material for: Study on the correlation between triglyceride glucose index, triglyceride glucose index to high-density lipoprotein cholesterol ratio, and the risk of diabetes in nonalcoholic fatty liver disease
Source: Front Endocrinol (Lausanne). 2025 Jun 23;16:1594548. doi: 10.3389/fendo.2025.1594548 (PMC12229875; doi:10.3389/fendo.2025.1594548)
Supplement: Supplementary file 6 [file Table5.docx]

Supplementary table 5. Association between TyG, TyG/HDL-c and the risk of diabetes in patients with NAFLD in the unbalanced dataset.

| Variables | Model 1 | | Model 2 | | Model 3 | |
| --- | --- | --- | --- | --- | --- | --- |
|  | OR(95% CI) | P | OR(95% CI) | P | OR(95% CI) | P |
| **TyG (continuous)** | 1.60(1.36-1.89) | <0.001 | 1.76(1.45-2.14) | <0.001 | 1.90(1.52-2.39) | <0.001 |
| **TyG (standardized)** | 1.92(1.54-2.41) | <0.001 | 2.20(1.68-2.87) | <0.001 | 2.44(1.78-3.34) | <0.001 |
| **TyG** |  |  |  |  |  |  |
| Q1 |  |  |  |  |  |  |
| Q2 | 1.59(0.73-3.48) | 0.243 | 1.61(0.72-3.58) | 0.247 | 1.61(0.71-3.69) | 0.255 |
| Q3 | 2.70(1.29-5.65) | 0.008 | 2.68(1.24-5.76) | 0.012 | 3.17(1.42-7.08) | 0.005 |
| Q4 | 4.76(2.35-9.66) | <0.001 | 5.70(2.59-12.55) | <0.001 | 6.37(2.68-15.16) | <0.001 |
| P for trend |  | <0.001 |  | <0.001 |  | <0.001 |
| **TyG/HDL-c (continuous)** | 1.60(1.34-1.92) | <0.001 | 1.68(1.36-2.07) | <0.001 | 1.68(1.34-2.10) | <0.001 |
| **TyG/HDL-c (standardized)** | 1.96(1.51-2.54) | <0.001 | 2.09(1.55-2.83) | <0.001 | 2.09(1.52-2.87) | <0.001 |
| **TyG/HDL-c** |  |  |  |  |  |  |
| Q1 |  |  |  |  |  |  |
| Q2 | 1.75(0.81-3.78) | 0.152 | 1.69(0.77-3.74) | 0.193 | 1.60(0.70-3.65) | 0.267 |
| Q3 | 2.31(1.10-4.88) | 0.027 | 2.46(1.13-5.36) | 0.024 | 2.87(1.26-6.55) | 0.012 |
| Q4 | 4.79(2.36-9.74) | <0.001 | 5.17(2.37-11.26) | <0.001 | 5.67(2.45-13.12) | <0.001 |
| P for trend |  | <0.001 |  | <0.001 |  | <0.001 |

^1^OR = Odds Ratio, CI = Confidence Interval; Model 1 : no covariates were adjusted; Model 2 : adjusted for Age, Sex, Marital, Smoking, Drinking, SBP, DBP, BMI, Hypertension, and CHD; Model 3 : adjusted for Age, Sex, Marital, Smoking, Drinking, SBP, DBP, BMI, Hypertension, CHD, TBIL, ALT, AST, Urea, CREA, UA, WBC, RBC, HB, and PLT.
